# Proximity to people with COVID-19 and anxiety among community residents during the epidemic in Guangzhou, China

**DOI:** 10.1192/bjo.2020.59

**Published:** 2020-07-23

**Authors:** Jinghua Su, Xiyuan Chen, Ning Yang, Meng Sun, Liang Zhou

**Affiliations:** Department of Social Psychiatry, The Affiliated Brain Hospital of Guangzhou Medical University (Guangzhou Huiai Hospital), China; Department of Social Psychiatry, The Affiliated Brain Hospital of Guangzhou Medical University (Guangzhou Huiai Hospital), China; Department of Social Psychiatry, The Affiliated Brain Hospital of Guangzhou Medical University (Guangzhou Huiai Hospital), China; Department of Social Psychiatry, The Affiliated Brain Hospital of Guangzhou Medical University (Guangzhou Huiai Hospital), China; Department of Social Psychiatry, The Affiliated Brain Hospital of Guangzhou Medical University (Guangzhou Huiai Hospital), China

**Keywords:** Psychological impact, new coronavirus, information overload, social media, anxiety

## Abstract

The recent outbreak of a novel coronavirus pneumonia (COVID-19) may have acute psychological consequences, both in relation to the impact of the virus itself and the restrictions imposed to tackle its spread. We conducted an online survey of 403 residents in Guangzhou, China. We found the prevalence of anxiety (defined as Generalized Anxiety Disorder–7 score ≥5) was 37.7%, and anxiety was significantly and moderately correlated with worry about COVID-19. Higher anxiety levels in community residents was associated with the presence of individuals with COVID-19 in the same building; a longer time spent each day gathering information about the virus; and choosing social media as their preferred source of information. Our findings provide an insight into the psychological support and guidance about information sources that are required in this type of public health emergency.

## Background

At the end of 2019 a novel coronavirus pneumonia (COVID-19) outbreak emerged in Wuhan, Hubei province, China, and spread rapidly to the rest of China and beyond, resulting in worldwide major public health concern.^1^ To stop transmission of the virus, urgent measures were put in place by the Chinese government,^2^ such as the prolongation of the Spring festival vacation, strict traffic control and the demand that the public stay at home. As the number of confirmed cases and deaths increased sharply and unprecedented isolation demands were imposed on the public there was an urgent need to examine the psychological impact on the public of the outbreak and the factors associated with it.

An outbreak of contagion usually has psychological consequences among the population. The 2003 outbreak of severe acute respiratory syndrome led to social disengagement, mental stress and anxiety, which subsequently led to a higher suicide rate in Hong Kong.^3^ A most recent survey in China during the COVID-19 epidemic revealed that the prevalence of depression and anxiety was 50.6% and 44.7%, respectively, among 1563 medical staff.^4^ However, little is known about the psychological impact on the public so far.

Community residents are required to follow a series of strict isolation demands, such as mandatory mask wearing, discontinuation of all forms of gathering and staying at home as far as possible. In the meantime, with the rise of the internet and smart phones, an enormous amount of information is flooding into people's lives through social media including for example WeChat, Weibo and Tik Tok. Timely reports about the epidemic can help the public to avoid areas where there are more people with the virus. However, information overload may be a risk factor for anxiety.

## Aims

The aim of this survey was to explore the prevalence of anxiety among community residents in Guangzhou city, the third biggest city in China, and its association with proximity to people with COVID-19, information overload and information sources.

## Method

### Participants and sampling

On 7 February 2020, there were 298 confirmed COVID-19 cases in Guangzhou. Data was collected from two housing compounds (KY and HL) that had newly confirmed cases in the 5 days before the survey, and one housing compound (DF) without any confirmed or suspected cases. Individuals were eligible to participate if they lived in one of the three selected communities, were aged 18 or above and had no family members diagnosed with COVID-19. One WeChat group of residents was selected in each housing compound. We distributed a non-transmissible link to the questionnaire in these three WeChat groups and invited group members to participate in the survey. The survey was conducted between 7 and 8 February 2020.

All participants had to read the informed consent and agree to participate before they could start the survey. This study was approved by the Institutional Ethics Committee of the Affiliated Brain Hospital of Guangzhou Medical University.

### Instruments

We collected data on participants’ demographic characteristics, proximity to COVID-19 cases, time spent collecting information about coronavirus, favourite methods to obtain information, worry about COVID-19 and anxiety. Proximity to COVID-19 was set as high (people with COVID living in the same building), medium (people with COVID living in the same housing compound but not in the same building) and low (no people with COVID in the same housing compound).

Time spent on collecting information about coronavirus referred to daily time used in collecting information relating to COVID-19, including television, newspaper, radio, websites, social media. A single item ‘In general, do you worry about the current situation of COVID-19?’ was used to evaluate the degree of worry. Four options were provided ranging from ‘very worried’ to ‘not at all’.

Generalized Anxiety Disorder (GAD-7), a brief self-rated scale validated in a Chinese population and commonly used in online surveys, was used to evaluate participant’ anxiety level in the past 2 weeks.^5,6^ There are seven items and the total score ranged from 0 to 21, with higher scores indicating severer anxiety. The total scores on the GAD-7 were categorised into no anxiety (0–4), mild anxiety (5–9), moderate anxiety (10–14) and severe anxiety symptoms (≥15).^7^

### Data analysis

Descriptive analyses were used to describe the demographic characteristics and prevalence of anxiety. Spearman's correlation was performed to probe the correlation between worry about COVID-19 and anxiety. Ordinal logistic regression was performed to explore factors associated with participants’ anxiety levels. All analyses were conducted using SPSS version 22.

## Results

A total of 1420 residents from three WeChat groups were approached, and 539 participants completed the questionnaire. In total, 136 were excluded because they were aged under 18, were not from the three sample housing compounds or had family members diagnosed with COVID-19. Finally, 403 participants were included in the analyses (response rate 28.4%). Twenty-eight participants who did not know that there were confirmed cases in their housing compound were classified into the group with no confirmed cases.

### Characteristics of participant

The mean age of the 403 participants was 42 (s.d. = 11.2) years, 68.5% were women, 87.3% were married and 79.7% had a college education or higher.

### Prevalence of anxiety

The mean score on the GAD-7 was 4.4 (s.d. = 4.8). The prevalence of anxiety was 37.7% (152/403); 23.1% had mild anxiety, 9.7% had moderate anxiety and 5.0% had severe anxiety. Significant and moderate correlation was found between worry about COVID-19 and GAD-7 scores (*r* = 0.545, *P* < 0.001).

### Multivariate ordinal logistic regression

Proximity to patients with COVID-19, time spent on collecting information about coronavirus and preferring to use social media as their information source were included in the multivariate analysis. Demographic characteristics including age, gender, marital status, education level and family income were also included. Table 1 shows that after controlling for demographic characteristics, the presence of cases of COVID-19 in the same building, spending more than 2 h each day collecting information about COVID-19 and preferring to use social media as their information source were significantly associated with anxiety, whereas no significant association was found between all demographic characteristics and anxiety.

**Table 1:** Multivariable analyses of the association between predictors and GAD-7 score in 403 community residents of Guangzhou city^a^

	*n* (%)	OR	95% CI	*P*
Proximity to people with COVID-19				
Same building	37 (9.2)	2.15	1.06–4.36	0.035
Same housing compound	228 (56.6)	1.28	0.82–1.99	0.282
No people with COVID in the housing compound	138 (34.2)	Reference	–	–
Daily time spent on collecting information related to COVID-19				
More than 2 h	96 (23.8)	1.84	1.04–3.23	0.035
1–2 h	172 (42.7)	1.28	0.81–2.01	0.281
<1 h	135 (33.5)	Reference	–	–
Choosing social media as their preferred source of information				
Yes	306 (75.9)	1.75	1.06–2.91	0.030
No	97 (24.1)	Reference	–	–

a. Ordinal logistic regression was performed adjusting for age, gender, marital status, education level and family income.

## Discussion

In this study, we focused on anxiety symptoms rather than on anxiety disorder because with anxiety symptoms interventions are possible at the community level. Anxiety was highly prevalent and is significantly correlated to worry about COVID-19 during the peak period of the COVID-19 outbreak. In a previous survey of 8917 community residents in Shanghai in 2014, only 7.8% reported GAD-7 scores of 5 or higher, much lower than that in our study.^8^ The moderate correlation between anxiety and worry about COVID-19 indicates that the elevated anxiety among community residents is likely a consequence of the COVID-19 epidemic. Anxiety was not associated with demographic characteristics in our study, which indicates the impact of the COVID-19 epidemic may be universal in community residents.

The presence of people with COVID-19 in the same building was associated with anxiety. This may be a reasonable emotional response to actual threat. There have been several news reports of multiple cases on different floors and in different apartments in the same buildings. Although individuals with confirmed/suspected cases were admitted to hospital immediately after being identified, more social and psychological support is needed for those who live in the same building.

Spending more than 2 h per day collecting information related to COVID-19, particularly through social media, was associated with anxiety. Our results are similar to findings from a previous study that indicated that post-traumatic stress is associated with indirect exposure to 9/11 through media.^9^ Social media often reports unconfirmed and/or unofficial news that may increase anxiety. Advice about an appropriate amount of time to spend collecting information through reliable sources should be given.

There are several limitations to our study. First, this is not a representative sample. Socioeconomic status is high among residents of the selected housing compounds. Hence, the prevalence of anxiety in our study is not generalisable. Second, we did not include other factors such as pre-existing mental disorders that may be related to anxiety. Third, the cross-sectional nature of this study prevents examination of causality. In conclusion, people who are experiencing higher levels of anxiety may spend more time collecting information about COVID-19.

## Data Availability

All data included in this study are available upon request by contact with the corresponding author.
